# Integrating Environmental Sensing into Cargo Bikes for Pollution-Aware Logistics in Last-Mile Deliveries

**DOI:** 10.3390/s25154874

**Published:** 2025-08-07

**Authors:** Leonardo Cameli, Margherita Pazzini, Riccardo Ceriani, Valeria Vignali, Andrea Simone, Claudio Lantieri

**Affiliations:** Department of Civil, Environmental and Material (DICAM) Engineering, University of Bologna, 40136 Bologna, Italy; leonardo.cameli2@unibo.it (L.C.); margherita.pazzini2@unibo.it (M.P.); valeria.vignali@unibo.it (V.V.); andrea.simone@unibo.it (A.S.); claudio.lantieri2@unibo.it (C.L.)

**Keywords:** environmental sensors, LCS, air quality, cycling routes, cargo bike

## Abstract

Cycling represents a significant share of urban transportation, especially in terms of last-mile delivery. It has clear benefits for delivery times, as well as for environmental issues related to freight distribution. Furthermore, the increasing accessibility of low-cost environmental sensors (LCSs) provides an opportunity for urban monitoring in any situation. Moving in this direction, this research aims to investigate the use of LCSs to monitor particulate PM2.5 and PM10 levels and map them over delivery ride paths. The calibration process took 49 days of measurements into account, spanning different seasonal conditions (from May 2024 to November 2024). The employment of multiple linear regression and robust regression revealed a strong correlation between pollutant levels from two sources and other factors such as temperature and humidity. Subsequently, a one-month trial was carried out in the city of Faenza (Italy). In this study, a commercially available LCS was mounted on a cargo bike for measurement during delivery processes. This approach was adopted to reveal biker exposure to air pollutants. In this way, the operator’s route would be designed to select the best route in terms of delivery timing and polluting factors in the future. Furthermore, the integration of environmental monitoring to map urban environments has the potential to enhance the accuracy of local pollution mapping, thereby supporting municipal efforts to inform citizens and develop targeted air quality strategies.

## 1. Introduction

The increase in the urban population has become an established trend, which, according to the United Nations Department of Economic and Social Affairs, will lead to approximately 70% of the global population living in urban areas by 2050. In Europe, the proportion is expected to reach 84%, corresponding to 599 million people [1]. These data inevitably imply an increase in the demand for urban mobility, particularly associated with socio-economic activities. In this context, the growing use of e-commerce for the purchase of goods has, in recent years, become one of the key factors significantly affecting traffic congestion and the quality of life in urban spaces [2]. The growth in e-commerce and last-mile deliveries has increased the complexity of urban transport systems [3]. Accordingly, recent studies have found that purchases made by private customers through e-commerce platforms account for 6% of the total daily incoming freight tons to cities and approximately 90% of the total number of deliveries carried out [4]. This steady growth in the urban population will systematically lead to an increase in greenhouse gas emissions as the demand for mobility, energy, and goods within cities will rise. In particular, the environmental impacts of freight movements are high in urban areas due to the densities of their populations [5].

In recent years, research on urban logistics models has been increasing [6]. Firstly, the European Commission has developed Sustainable Urban Logistics Plans (SULPs), complementary to the Sustainable Urban Mobility Plans (SUMPs), for cities facing logistics challenges [7]. In particular, the integration of cargo bicycles in freight transport can contribute to the achievement of this goal [8]. Particularly in large cities, besides environmental benefits, eco-friendly vehicles introduce many advantages such as lower operating costs, higher flexibility, and reduction in the time needed for loading/unloading operations [9]. Therefore, despite the positive aspects from sustainable urban logistic transport, many nations lack the preparedness to design an effective and specific strategy [10]. This is fundamental if were are to fully understand the potential of this operation, enabling an understanding of its hard constraints and benefits/costs. Given the promising advantages of cycle logistics, the scientific community has initiated various pilot projects worldwide to evaluate their positive effects and limitations in real-world scenarios. In Bologna, the URBANE project has been testing a last-mile logistics model based on a network of three microhubs located along the city’s ring roads. Preliminary results indicate that this model reduces van travel distances within the historic core by approximately 50% [11]. This project has provided an important opportunity to validate the introduction of cargo bikes and micro-depots for delivery companies. Field tests in Stargard [12] revealed a fragmented and insufficient cycling infrastructure, often forcing cyclists to merge with motor traffic in unsafe conditions. Despite this, cargo bike deliveries were feasible and positively received by both drivers and pedestrians. The pilot project suggests that, in urban areas characterized by poorly developed infrastructure, lighter and more maneuverable cargo bikes are more suitable for ensuring operational efficiency and rider safety. These two highlighted projects do not present any specific analysis focused on environmental impact. On the contrary, other studies have demonstrated significant environmental benefits. In Seattle [13], an e-cargo tricycle pilot, involving multiple stakeholders, used delivery data to compare performance with traditional cargo vans. The results showed that microhubs and e-cargo cycles can reduce the vehicle miles traveled and emissions. However, the benefits depend heavily on efficient upstream logistics when restocking the microhubs. A participatory design approach offers insights into future implementation, highlighting the importance of stakeholder engagement. In addition to the pilot project tested in the field, simulation studies can evidence key aspects. Research in Berlin [14] has evaluated simulation results for integrating micro-depots and e-cargo bikes for last-mile deliveries into urban logistics networks. It showed significant reduction in environmental impacts (80% greenhouse gas emissions) and total delivery distance (65.9% reduction in total mileage) as a result of replacing diesel vans. These findings support the promotion of micro-depots and e-cargo bikes. The results highlight environmental savings due to the reduced vehicle kilometers traveled. However, these were not directly related to or assessed against air quality levels in the specific context of analysis.

More recently, attention has been focused on the challenges and key factors influencing the integration of micromobility into last-mile deliveries. The new construction and maintenance of bicycle infrastructure has encouraged the use of cargo bicycles, making them attractive for new emerging companies [15]. The width and number of lanes and the interaction between cargo bikes and motorized vehicles can strongly influence rider safety [16] and traffic accidents [17,18]. A more complete bike network is needed to accommodate the future expansion of cargo bike operations, and more effort is required to improve the bike accessibility of distribution centers [19,20]. Additionally, local authorities often lack the capacity and expertise required to implement effective freight decarbonization strategies. This institutional gap is further complicated by the role of public perception. Citizens’ views on urban infrastructure and environmental concerns can significantly influence the success of such measures, emphasizing the need to consider social acceptance in the formulation of urban logistics.

For this reason, the environmental measures that provide information, not only on emissions savings but also on the actual air quality, could raise public awareness [21]. This would assign a dual purpose to cargo bikes for last-mile delivery: promoting both operational efficiency and the improvement of urban health [22]. In recent years, low-cost sensors (LCSs) have emerged as complementary tools of conventional monitoring equipment, enable more effective air quality assessment [23]. They significantly enhance the spatial resolution of air monitoring data and foster citizen engagement in experimental measurement campaigns [24]. Given the wide availability of low-cost sensors [25], it is possible to quantify urban pollution at street level. Standards for evaluating performance of low-cost particulate matter (PM) sensors are not yet established, and manufacturers often provide limited performance data [26]. Additionally, data from LCSs are susceptible to inaccuracies caused by long-term sensor drift, calibration difficulties, and sensitivity to temperature and humidity variations [27]. Indeed, presenting readings from these types of sensors without proper calibration and accompanying data-quality metrics may lead to misleading interpretations [28]. In response, researchers have begun addressing this gap by systematically assessing the accuracy and reliability of various PM sensors under several environmental conditions. Especially, crowd-sourced data from multiple LCSs provided by different companies were extensively studied [29]. The literature reveals a lack of assessment concerning the routes used by cargo bike riders.

For these reasons, this research aims to integrate the use of LCSs by testing a prototype for random cargo-bike delivery routes. Specifically, Section 2 contains detailed information on the used sensors, obtained data, calibration process, and project structure, as well as a flowchart to provide visual clarification. Section 3 shows and critically discusses the results of the calibration. The final section lays the groundwork for future developments in this area. This approach can provide valuable insights into the environmental quality of urban routes. In doing so, beyond reducing the emissions associated with urban logistics, the project seeks to provide cargo bike riders, and subsequently the broader public, with a mapping of routes that are less impactful in terms of particulate matter concentration. Findings emerging from this research have important implications for improving courier health, as they provide delivery companies with the opportunity to plan routes based on less-polluted paths, reducing riders’ exposure to air pollutants during their operations. Moreover, these results can support the development of sustainable urban planning policies by offering valuable data without requiring additional fieldwork. Ultimately, similar projects could demonstrate a scalable methodology that is capable of being applied across urban contexts to connect environmental monitoring and the sustainability of last-mile logistics.

## 2. Methods

**Figure 1:**
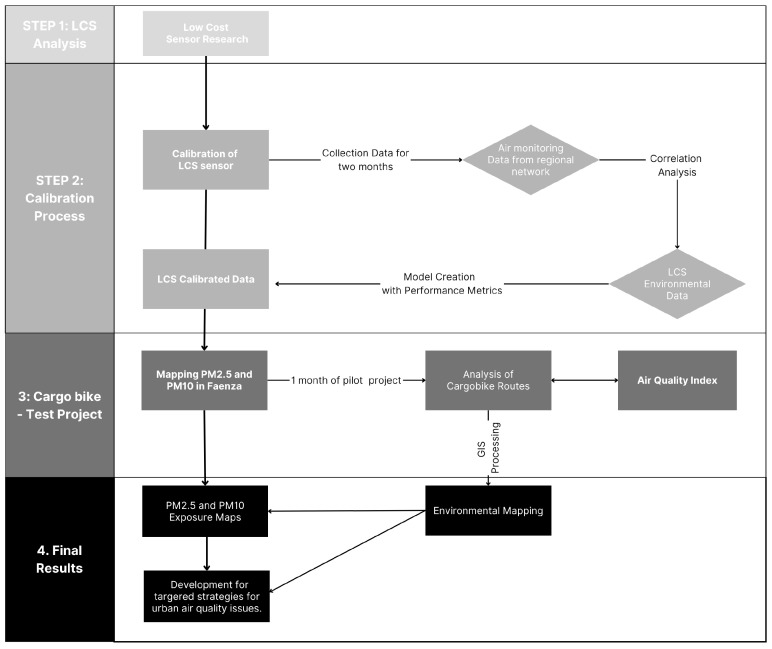
Flowchart of research activities.

The research aims to evaluate cargo bike delivery routes from a health-related perspective by focusing on exposure to particulate matter (PM2.5 and PM10) during morning delivery operations. The complete flowchart of activities and operations, encompassing calibration process, test field, and results, is reported in the figure below (Figure 1).

The present study is embedded within the Faenza project “Transformation Of Goods Logistics In The Romagna Faentina Union” [30], which promotes sustainable last-mile logistics through cargo bikes and air quality monitoring. The pilot project lasted one month. For this reason, mobile low-cost sensors (LCSs) were selected for air quality monitoring. This type was chosen for its compact size, dual PM-detection capability, GPS integration, and real-time data logging [31]. While LCSs are less precise than fixed monitoring stations, their mobility enables high-resolution spatial data collection. For this reason, to ensure data accuracy, the sensors were calibrated by comparing them with fixed stations, using around 2 months of data, followed by applying statistical correction models. In particular different regression models were tested and evaluated through model validation metrics (R^2^, RMSE, and MAE). This calibration enabled the reliable mapping of exposure levels along different delivery routes. The data from the LCSs were collected during the morning deliveries. GPS data were then processed, through GIS software, to create paths based on their pollution exposure levels. This makes it possible to layer the foundations for route optimization that prioritize rider health and urban air quality. The following paragraphs will provide a detailed overview of the sensors, calibration methods, and the environmental index used in the aforementioned project.

### 2.1. Mobile Sensors

The development of increasingly advanced technologies for creating low-cost, ever-smaller environmental sensors has ushered in a new era of environmental monitoring [32]. These sensors can be used for two main purposes: primarily as input parameters for air quality monitoring and to validate environmental exposure models [33]. Furthermore, according to the World Meteorological Organization (WMO) [34], LCSs are becoming tools of paramount importance in tackling the challenge of addressing gaps in air quality monitoring networks at both the global and local levels. These devices are flexible and can provide valuable data to support policy strategies that are aiming to improve air quality. The concept of ‘low cost’ refers mainly to the unit cost, which is significantly lower than conventional reference tools. However, these cost savings inevitably entail technical compromises, such as lower data quality, selectivity and sensitivity to low concentrations, as well as reduced resistance to high concentrations and low device lifetime. As matter of fact, the validation of obtained measures from selected sensors in the case study has to be underlined as a step of paramount importance.

For academics engaged in this type of environmental particulate analysis, the literature provides some guidelines for selecting the most appropriate sensors. It was highlighted that air quality data, collected by LCSs, have the potential to be publicly accessible, informing the community about the air quality in their area [35]. LCSs used to monitor atmospheric particulate matter (PM) typically operate based on the principle of light scattering or by means of image processing methods [36]. Light scattering is the process by which an electromagnetic wave (incident light wave) encounters an obstacle (such as a particle) and is deflected along a new trajectory. Light is generally deflected by any heterogeneity present in the illuminated medium. Light scattering technique is particularly well-suited to inexpensive devices as it enables the production of LCSs with low power consumption and fast response times [37]. These characteristics make them ideal for large-scale applications where efficiency and affordability are fundamental. Despite their proper functioning, LCSs have inherent limitations in terms of sensitivity and lifespan. To address these issues, the present study employed two different sensors to evaluate the measurements and established a calibration procedure, which is detailed in Section 2.3.

**Figure 2:**
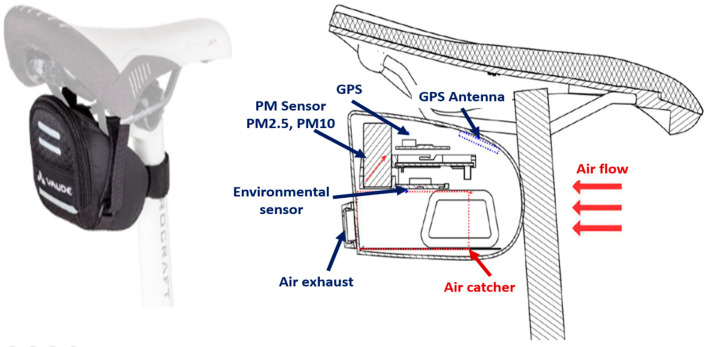
Smart Citizen Kit concept.

The sensor package provided by the University of Bologna consists of a Smart Citizen Kit [31] with a GPS, antenna, and a case that attaches to the underside of the seat with straps. The Smart Citizen Kit is a complete set of modular hardware components detigned with the aim of providing tools for environmental monitoring, ranging from ‘citizen science’ to the most advanced scientific research. The environmental sensors are enclosed in the saddle bag to prevent dirt and water infiltration, while still enabling the measurement of the pollutants listed in the table below. The kit is rechargeable via a USB cable connected to a power socket. All the measurements were obtained using two different sensors; their details are reported in Table 1 and visually shown in Figure 2. It should be noted that this study focused on temperature, humidity, and particulate matter measurements. Therefore, once the sensor for this study was identified, the calibration method for validating the collected measurements was established.

### 2.2. Calibration Process

The primary stage in the investigation of the sensor’s aptitude for smart environmental monitoring was the comparison of the obtained data from the mobile sensor with open-source data from fixed air quality monitoring stations in the Municipality of Bologna. The fixed sensor used as a reference is located in the city of Bologna, belonging to a regional agency that monitors environmental indicators (including particulate levels). To obtain a consistent dataset for comparison purposes, cycle trips were undertaken within two different time slots: morning (7:00–8:00 a.m.) and afternoon (17:00–18:00 p.m.). These spanned 55 working days from May 2024 to November 2024. The seasonal differentiation was chosen with the aim of monitoring data under different environmental conditions and seasons [38]. However, due to sensor malfunctions and missing data from the fixed station, the final dataset comprises 49 days (*n* = 49). The data obtained from the fixed sensor are furnished as the daily average. Conversely, the data provided by the mobile sensors are characterized by a resolution of one data point every five seconds. Therefore, to facilitate a meaningful comparison between the two datasets, a daily average was used as a reference unit scale. Subsequently, various statistical methods were applied for calibration. Based on the literature, different models were considered to correct the collected data from the mobile sensors; specifically, multiple linear regression and robust regression were evaluated. Both models were validated using metrics including R^2^, RMSE, and MAE. In conclusion, based on these parameters, supported by multicollinearity and heteroscedasticity analyses, the sensors’ data were re-calibrated. Section 3 provides a detailed description of this procedure.

### 2.3. Air Quality Index

The raw data obtained from LCSs often lack accuracy and reproducibility in comparison with the data from the fixed sensor [26,29]. Therefore, relying on LCSs alone for precise measurements requires careful calibration and data processing. In this research, the data gathered along the cycling routes were accordingly processed and interpreted in relation to the established air quality standards, defined by the current regulations. The pollutants analyzed in this study are particulate matter, specifically PM2.5 and PM10 (airborne particles with diameters less than or equal to 2.5 and 10 μm, respectively). PM2.5 levels in urban environment can vary significantly depending on factors such as traffic density, industrial activity, meteorological conditions, and the presence of other pollution sources.

However, guidelines and standards established by international and national organizations exist to classify PM2.5 levels in terms of air quality. According to European regulations on the matter [39], air quality is assessed based on PM2.5 and PM10 concentration thresholds. It is important to underline that Italian air quality legislation [40] does not provide a classification of exposure levels to particulate matter based on thresholds. Instead, it establishes fixed legal limit-exceedance values. Specifically, for PM10, the regulation sets a daily limit value of 50 µg/m^3^, which must not be exceeded more than 35 times per year, and an annual limit value of 40 µg/m^3^ (Table 2a). For PM2.5, only an annual limit value is defined, set at 25 µg/m^3^. Therefore, to calculate the air quality index on a cycling route, the data were pre-processed, averaged to an index composed of 4-value ranges (Table 2b) based on the World Health Organization (WHO) classification and the relevant body of literature on the subject [23,41]. In conclusion, as case study, a pilot project was conducted to measure the sensors’ performances.

### 2.4. Urban Logistic Transformation in Faenza (Italy) Pilot Project

To evaluate the applicability of the above-described methodology, a study area was selected the urban center of Faenza City, located in Emilia-Romagna Region (Italy). The pilot project aimed to identify strategies for reducing air and noise pollution caused by urban freight distribution in the city center. The core intervention of the research involves limiting the access of commercial vehicles to the central area, regulated by a limited traffic zone, and accomplishing last-mile deliveries using zero-emission vehicles stationed at strategically located urban logistics hubs.

In order to understand the dynamics of freight distribution within Faenza’s center, a complete mapping of all commercial activities was conducted in precedent step. Following this, a questionnaire was distributed to collect detailed information regarding goods procurement processes. Notably, 40% of respondents reported that deliveries were made using irregular parking practices, with vans stopping outside of dedicated public or private loading bays. In this context, the adoption of smaller delivery vehicles, such as cargo bikes, can help reduce the total time of occupancy at loading/unloading zones and discourage illegal parking practices.

The pilot experiment, which ran for one month (from 17 June to 17 July, with 19 days of data collection) in the morning (between 8:00 a.m. and 1:00 p.m.), involved the use of random cargo bike routes in conjunction with hybrid diesel vehicle routes. The duration of the pilot was agreed upon with the stakeholders, primarily based on the availability of the microhub space. Furthermore, several studies in the literature suggest that pilot project durations can vary significantly, ranging from just a few days [41] to several months [9], depending on the objectives and operational constraints. In this case, the collected data serve as a valuable starting point for building a comprehensive understanding of performance and scalability.

**Figure 3:**
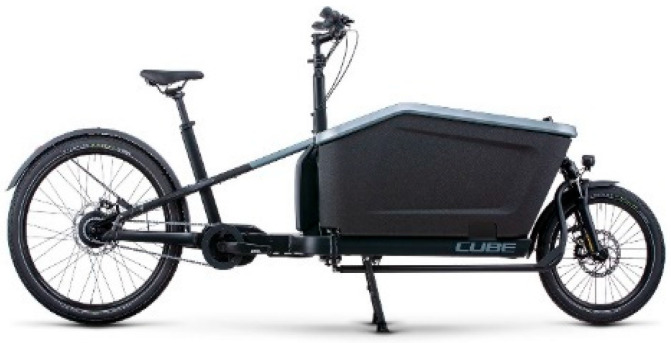
Cargo bike of the Faenza Pilot Project.

A logistics hub was set up for the transshipment of goods from the traditional van to the cargo bike. The cargo bike (Figure 3) used in the experiment has a maximum payload capacity of 220 kg and is able to transport bulky items with a volume of up to 200 L. It is a pedal-assisted bicycle, with an engine located at the base of the steering tube, and it is equipped with a compartment near the center of gravity to accommodate up to two batteries. These batteries provide enough power for a full 8-h working day and are recharged overnight to be ready for the following day’s deliveries.

In the following sections, the data collected from the environmental sensors will be analyzed; the sensors were properly installed on the cargo bikes. These data were used to assess the spatial coverage of the deliveries, the routes actually undertaken, and the corresponding environmental mapping of cycle trajectories.

## 3. Results

As previously discussed, one of the primary objectives was to verify the relationship between the data from a mobile environmental sensor and those from the reference station. In order to establish a basis for comparison, the correlation between the two measures of PM2.5 and PM10 values was initially analyzed. The results indicated a high linear correlation between the two measures. Subsequently, a specific multiple linear regression (MLR) (cap 3.2) approach was used to investigate the factors influencing the discrepancy between the daily averages of both pollutants. MLR is widely used as statistical tool to explain part of the observed variability through a set of regressors and the interactions between them. The greater the explained variability, the more accurate the prediction [42]. MLR is based on the following equation (1):

(1) $$y_{i}=\beta_{0}+\sum_{j=1}^{p}x_{ij}\beta_{j}+\varepsilon_{i}$$

where y_i_ is the dependent variable; β_0_ is the constant; β_j_ is the coefficient associated with each regressor, x_ij_; ε_i_ is the random error term. The following table (Table 3) provides a comprehensive overview of all the variables that were incorporated into the aforementioned analysis.

### 3.1. Correlation Results

The Pearson’s correlation matrix presented in Table 4 (*n* = 49), shows the correlation coefficients between all the variables under analysis.

The results demonstrate how PM2.5 and PM10 levels coming from the mobile sensors exhibit perfect correlation (r = 1.000, *p* < 0.01), reflecting strong internal consistency between the two particulate types. In the same way, PM2.5 and PM10 gathered from the fixed sensor show a very strong correlation (r = 0.938, *p* < 0.01). The coefficients also highlight strong cross-correlations between particulates’ levels coming from the two sensors under consideration. The following observations are worth noting:

- Mobile_Sensor_PM2.5 is strongly correlated with both Fix_Sensor_PM2.5 (r = 0.891, *p* < 0.01) and Fix_Sensor_PM10 (r = 0.784, *p* < 0.01).
- Mobile_Sensor_PM10 exhibit (r = 0.893, *p* < 0.01) with Fix_Sensor_PM2.5 and (r = 0.785, *p* < 0.01) with Fix_Sensor_PM10).

These last results further enhance the correlation between the two types of particulates even if they are obtained from two different sources, validating the accuracy of mobile sensors in environmental measurement contexts comparable to fixed stations. In coherence with previous research [43,44,45], temperature and humidity also exhibit a notable degree of correlation with particulate concentration. Temperature is positively and significantly correlated with all particulate matter measurements, with stronger correlations observed for mobile sensors (r = 0.731 and 0.734) compared to fixed ones (r = 0.655, 0.505), all significant at the *p* < 0.01 level. The results also demonstrate a strong positive correlation between temperature and humidity (r = 0.794, *p* < 0.01), meaning that an increase in temperature is associated with an increase in humidity levels. Humidity also shows significant, albeit slightly weaker, correlations with particulate matter levels. The mobile sensor readings correlate moderately with humidity (r = 0.553 and 0.591, *p* < 0.01), whereas fixed sensor correlations are lower (r = 0.454, *p* < 0.01 for PM2.5; r = 0.308, *p* < 0.05 for PM10).

**Figure 4:**
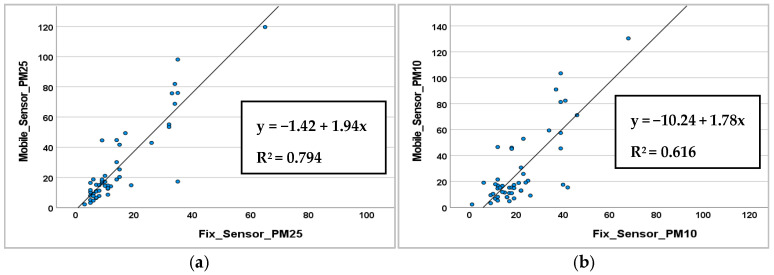
Correlation analysis for PM_2.5_ (**a**) and PM_10_ (**b**).

Subsequent to the correlation matrix, to identify and utilize linear or nonlinear tendencies between variables (the two pollutants in this case) as well as outliers, a scatterplot was employed. Figure 4 shows the two scatterplots for the PM data, gathered using the two types of sensors (fixed and mobile).

**Figure 5:**
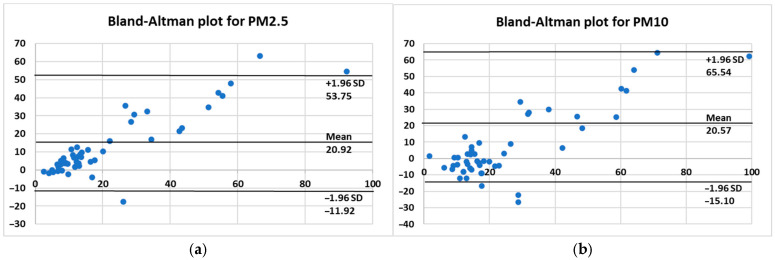
Bland–Altman plot for PM_2.5_ (**a**) and PM_10_ (**b**).

Regarding the PM2.5 particulate, the linear R^2^ value of 0.794 indicates a strong linear correlation between the values, specifically meaning that around 79% of the variance is explained by the fix sensor. For particulate PM10, the linear R^2^ value is 0.616, indicating a lower degree of linear correlation although the value remains high. For both graphs, the angular coefficients are >1, meaning that the mobile sensor tends to overestimate the particulate level. In conclusion, the analysis reveals a robust correlation between the two stations, as evidenced by the coefficient of determination. Nevertheless, correlation alone is insufficient for claiming interchangeability between the two instruments, as a high correlation can coexist with significant systematic differences. To this end, the analysis was supplemented with the Bland–Altman method (Figure 5), which quantifies the average bias between the two measurements and evaluates their dispersion. For PM2.5, the mean bias is 20.92 µg/m^3^, with concordance limits ranging from −11.92 µg/m^3^ to 53.75 µg/m^3^. These results confirm the presence of a systematic overestimation by the mobile control unit but also indicates that this difference is relatively stable across the measurement range.

In the case of PM10, the correlation is weaker (R^2^ = 0.616) and the regression line has a slope of 1.78, indicating an overestimation by the mobile monitoring station but with greater variability. The Bland–Altman analysis demonstrates a comparable average bias to that of PM2.5 (20.57 µg/m^3^), yet it exhibits wider limits of agreement (−15.10 to 65.54 µg/m^3^), indicating a diminished degree of comparative reliability between the two instruments for this particular parameter.

In summary, the integration of correlation and Bland–Altman allows us to distinguish between linear relationship and actual agreement. While correlation highlights a consistent trend between measurements, Bland–Altman reveals that there is a systematic positive bias in both cases, which is more moderate and stable for PM2.5 and more variable for PM10. Therefore, although the two monitoring stations can be considered to be consistent for PM2.5 monitoring (possibly applying a systematic correction), the use of PM10 data requires greater caution and, possibly, more thorough calibration.

This overestimation can be either related to different calibration systems of the two devices or surrounding conditions which influence the concentration of particulate pollutants. In this regard, it has to be mentioned that the two sensors are affected from a different acquisition height and this variable is known in the literature to influence particulate concentration [46]. For instance, ref. [47] found that, in urban environments, particle concentrations (specifically PM10 and PM2.5) decrease by approximately 37% and 35%, respectively, when measured from ground level up to 14 m. In the case study presented in the article, the fixed sensor is located in open-air conditions at a height of around 2 m, while the mobile sensor’s location and condition changes according to site-specific characteristics, and is positioned at a height of 0.75 m.

### 3.2. Model Performance

The strong linear correlation, obtained in paragraph 3.2, demonstrates the validity of MLR as analysis tool (cap. 3.2.1 and cap. 3.2.2). As previously mentioned, two different MLR measurements have been carried out in order to calibrate the sensors’ data with those from the reference station in the Municipality of Bologna. Additionally, a robust regression was used to support our understating of which model fits better (cap. 3.2.3). In this specific application, the model regressors used were the data on the humidity, temperature, and pollutants (PM2.5 and PM10) coming from the fixed sensors. In an initial attempt, distance from the two sensors was also considered, but unacceptable values were obtained in the Pearson’s correlation.

#### 3.2.1. Multiple Linear Regression for PM2.5

An MLR analysis was conducted to evaluate the effect of five independent variables (Mobile_Sensor_PM25, distance, precipitation, humidity, and temperature) on the dependent variable, Fix_Sensor_PM25. The model results (Table 5) were shown to be statistically significant (F = 63.853, *p* < 0.001), showing their explanatory power and an R^2^ value of 0.810, as indicated by the ANOVA (Table 6).

The results indicate that the model is statistically significant in explaining the variance of the dependent variable, so 81% of its variance is explained by the included predictors. The adjusted R^2^ = 0.797 is slightly lower than the R^2^, highlighting the robustness of the model. Durbin–Watson (DW = 1.989) indicates the absence of a significant autocorrelation in the residuals. The sum of squares that are attributable to the regression (5904.492) accounts for the majority of the total sum of squares (7291.551), highlighting the effectiveness of the model.

As expected, the most relevant predictor is Mobile_Sensor_PM25 with a standardized coefficient Beta = 0.891 that indicates a strong positive effect, meaning that an increase in the standard deviation of 1 in the mobile sensor measurement leads (on average) to an increase in the standard deviation of 0.891 in the Fix_Sensor_PM25. Furthermore, the unstandardized coefficient B = 0.410 (*p* < 0.001) supports the practical significance of this finding by suggesting that a unitary increase in PM2.5 values gathered by the mobile sensors is associated with an average 0.41 µg/m^3^ increase in fixed sensor measurements. Those results, in coherence with the data obtained for the correlation analysis, tends to confirm the strong consistency between the two devices and the validity of mobile sensors as complementary measuring instruments [41].

Humidity, contrary to the previous predictor, demonstrates a negative standardized Beta coefficient equal to −0.208, thus indicating an inverse effect: as relative humidity increases, there is a slight decrease in PM2.5 levels, as measured by the fixed sensor. This effect was found to be marginally non-significant (*p* = 0.058), However, it did approach the conventional threshold of 0.05. The negative Beta value indicates that an increase in humidity, measured in standard deviations, is associated with a reduction in the estimated concentration of fine particulate matter. In non-standardized terms, a one percentage point increase in humidity would result in an average decrease of 0.281 µg/m^3^ in PM2.5 concentration. The negative standardized coefficient (Beta = −0.168) is also demonstrated for temperature, indicating a theoretically inverse yet non-significant effect (*p* = 0.190). Despite the non-significance of the effect, temperature has been kept as an independent variable due to its strong correlation with PM2.5, as shown in paragraph. 3.1, and because of its strong significance which has been attested to in the literature [48]. The non-standardized value (B = −0.297) indicates that an increase of 1 °C is associated, on average, with a reduction of approximately 0.30 µg/m^3^ in the fixed sensor values.

All the collinearity indicators (VIF < 4) fall within the acceptable limits, ruling out multicollinearity issues between the predictors. VIF values below 4 are widely regarded in the literature as indicating low multicollinearity. This threshold is therefore consistent with the commonly accepted methodological standards [49]. Overall, the model exhibits favorable statistical properties.

From the coefficients, it is possible to outline the MLR equation, which is reported below (2):

(2) Fix_Sensor_PM25=28.027+0.41Mobile_Sensor_PM25−0.297Temperature−0.208Humidity

**Figure 6:**
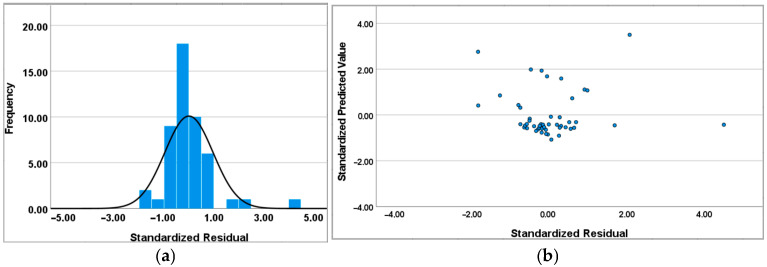
Histogram (**a**) and scatterplot (**b**) to standardize the residual PM2.5.

As shown in Figure 6, the standardized residuals are visualized both as a histogram and as a scatterplot against the standardized predicted values. This dual representation serves to assess two key assumptions of linear regression: the normality of residuals and the homoscedasticity of their variance. The approximately Gaussian distribution of the residuals (Figure 6a) supports the assumption of normality, which is essential for the validity of inferential statistics such as *p*-values and confidence intervals. Simultaneously, the absence of discernible patterns or systematic structures in the scatterplot (Figure 6b) indicates that the residuals are randomly distributed around zero, suggesting homoscedasticity and confirming that the model does not suffer from major specification errors. Together, these diagnostics provide strong evidence that the model offers a statistically sound fit to the data [50].

#### 3.2.2. Multiple Linear Regression for PM10

By following the same path for the precedent particulate type, for PM10, an MLR analysis was conducted by considering the same set of variables (Mobile_Sensor_PM10, distance, precipitation, humidity, and temperature) on the dependent variable, Fix_Sensor_PM10: see Table 7. The model’s results are slightly lower in statistical significance in comparison with the previous case (F = 28.372, *p* < 0.001), and achieved an R^2^ value of 0.645, as indicated by the ANOVA (Table 8). The adjusted R^2^ = 0.797 is again slightly lower than the R^2^, confirming the robustness of the model. The results of the Durbin–Watson (DW = 1.519) indicated no sever autocorrelation in the residuals. The sum of squares attributable to the regression (5134.689) accounts for the majority of the total sum of squares (7849.347), highlighting the effectiveness of the model.

The outcome of regression model, constructed to explain the PM10 values measured by the fixed sensor, confirms the paramount role of the mobile sensor as the main predictor, suggesting again the strong correlation between the two instruments; as matter of fact, the standardized coefficient Beta = 0.902 again indicated a strong positive relationship. On the other hand, the unstandardized coefficient (B = 0.398, *p* < 0.001) serves to reinforce the practical implication, which suggests an expected increase of 0.398 µg/m^3^ for each additional µg/m^3^ detected by the mobile sensor.

These results are fully consistent with those observed in the PM2.5 model, where Mobile_Sensor_PM25 exhibited a comparable Beta (0.891) and a B coefficient of 0.410. In both cases, the data demonstrate a strong alignment between the mobile and fixed sensors, with very high significance values (*p* < 0.001), thus confirming the quality of the measurements made by the mobile devices for both particulate size fractions.

A more pronounced divergence in the analysis of meteorological variables was observed. In the PM2.5 model, humidity exhibited an almost significant effect; instead, in the MLR applied to PM10, this effect is more pronounced in terms of Beta (−0.271), but still not completely significant (*p* = 0.067). However, its significance at the level of 90% was confirmed.

Temperature exhibited a negligible effect in both models, with a Beta of −0.168 (*p* = 0.190) in the PM2.5 model and −0.058 (*p* = 0.736) in the PM10 model. In both cases, the effect is assessed to be not statistically significant; however, the same considerations were made before applying it to this case. The VIF statics fall in the same range (VIF < 4), indicating no demanding collinearity issues. In the same way, the MLR equation obtained from the table of coefficients is also reported for PM10 (3):

(3) Fix_Station_PM10=36,522+0.398Mobile_Sensor_PM25−0.106Temperature−0.380Humidity

**Figure 7:**
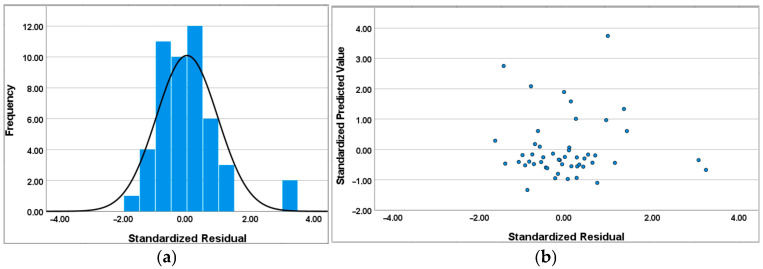
Histogram (**a**) and scatterplot (**b**) for standardizing the residual PM10.

For the residual analysis (Figure 7), the same considerations as those mentioned before can be applied to the MLR for PM10.

#### 3.2.3. Model Evaluation

For comparing the multiple regression model, a RANSAC robust regression (RR) was conducted by python code, considering the same set of variables. RR falls within a class of statistical methods that were specifically developed to produce reliable estimations of the presence of outliers or deviations. RR aims to reduce the influence of anomalous data, thereby improving the stability and the accuracy of the estimated coefficients [51]. Among these techniques, the Random Sample Consensus (RANSAC) algorithm is commonly used. It operates by iteratively fitting models to randomly selected data subsets and evaluating their performance based on the largest set of consistent observations (inliers). However, a limitation of RANSAC is that it does not natively support statistical inferences such as the estimation of standard errors, t-statistics, or *p*-values, because it only fits the final model to a subset of the data. Its use ensures that model predictions remain valid even in challenging data conditions, such as quality analysis, transport studies, and geospatial data modeling. For this reason, the resulting RR Equations (4) and (5) are as reported here:

(4) $$Fix_{StationPM2.5}=28.02+0.41Mobile\_Sensor\_PM2.5-0.297Temperature-0.281Humidity$$

(5) $$Fix_{StationPM10}=37.53+0.429\;Mobile\_Sensor\_PM10-0.127Temperature-0.392Humidity$$

**Figure 8:**
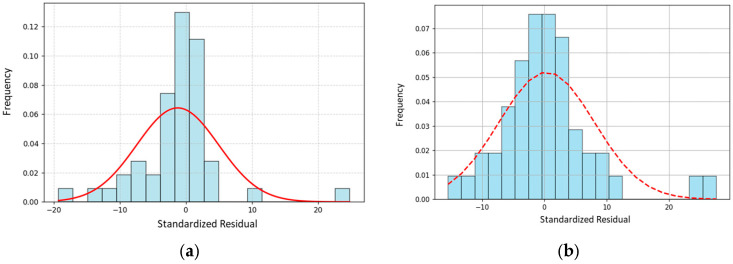
Histograms for standardized residuals of PM2.5 (**a**) and PM10 (**b**).

The histogram of standardized residuals for PM2.5 (Figure 8a) shows a roughly symmetric distribution around zero, but with noticeable deviations from normality in the tails, indicating some outliers or heteroscedasticity. The red curve (normal distribution) suggests that the residuals are slightly skewed and not perfectly Gaussian.

The histogram of the standardized residuals for PM10 (Figure 8b), compared with the overlaid normal distribution curve, indicates that the residuals are approximately centered around zero, suggesting no substantial bias in the predictions. However, the presence of heavier tails and slight deviations from the bell-shaped curve suggests potential outliers or mild departures from normality. While such deviations do not necessarily invalidate the model, they may slightly affect the reliability of parametric significance tests, warranting further diagnostic checks.

To evaluate the effectiveness of each calibration strategy, several performance metrics have been computed [52], namely the coefficient of determination (R^2^), the mean absolute error (MAE), and the root mean squared error (RMSE). Those are summarized in Table 9.

Both modeling approaches exhibit similar coefficients of determination (R^2^), with 0.730 for PM2.5 and 0.630 for PM10, indicating that they explain a similar proportion of variance in the data. However, differences emerge in error metrics. The linear regression model yields slightly lower RMSE values compared to the robust regression, particularly for PM10, where the RMSE increases from 7.44 to 7.77. The regression equations highlight that the robust model assigns higher weights to the mobile sensor PM2.5 variable while attenuating the influence of temperature and humidity. This shift suggests that robust regression adapts to outliers by adjusting coefficient estimates, resulting in a lower intercept for PM2.5 compared to the linear model. For PM10, the differences in coefficients are marginal, implying that the linear regression estimates were relatively stable and less affected by outliers.

In addition, a cross-validation was performed for the RR. Regarding the PM2.5 results, the 5-fold cross-validation results indicate a slightly better performance of the multiple linear regression model compared to the robust RANSAC approach for the PM2.5 calibration. The linear regression achieved a mean R^2^ of 0.73 (±0.19) with an RMSE of 5.84 µg/m^3^ (±2.17), whereas the RANSAC model reported a mean R^2^ of 0.64 (±0.21) and an RMSE of 6.55 µg/m^3^ (±2.07). These results suggest that approximately 73% of the PM2.5 variability is explained by the linear model, which slightly outperforms RANSAC in terms of both the explained variance and the error metrics. The relatively close performance of the two models also indicates that the dataset is not significantly influenced by outliers, making the standard linear regression a robust and reliable choice for this calibration task.

The 5-fold cross-validation results indicate comparable performance between the multiple linear regression and the robust RANSAC model for PM10 calibration. The linear model achieved a mean R^2^ of 0.51 (±0.31) with an RMSE of 7.91 µg/m^3^ (±2.88), while RANSAC reported a mean R^2^ of 0.50 (±0.33) and an RMSE of 8.10 µg/m^3^ (±2.96). These findings suggest that approximately 50% of the PM10 variability is explained by both models, with the linear regression slightly outperforming RANSAC in terms of accuracy. However, the similar results highlight that the dataset is not strongly affected by outliers, and both approaches provide stable and reliable calibration performance.

### 3.3. Bicycle Routes

**Figure 9:**
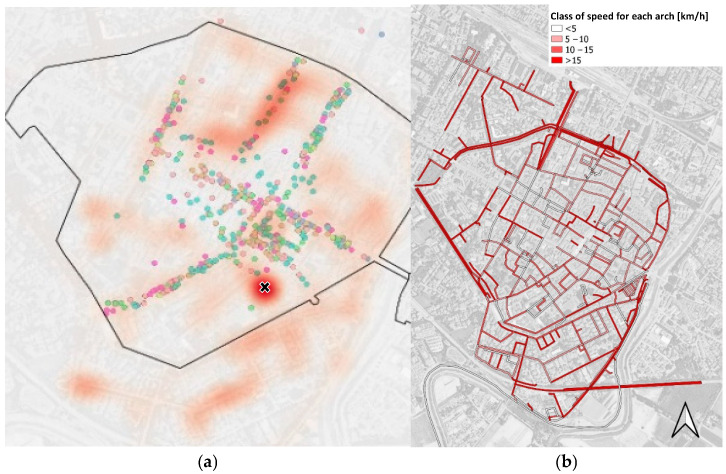
Delivery heatmap (**a**) and speed (**b**) during delivery process.

The routes, tracked via GPS integrated into the sensor mounted on the cargo bike, were analyzed using Q-GIS (v.3.34.9) software. The input parameters are representative of the measurements collected by the sensor, including temperature, humidity, and particulate matter concentrations. Geolocation was provided by the sensor system and referenced to the WGS84 Pseudo-Mercator coordinate system (EPSG: 3857). The reconstruction of the daily delivery routes was based on the recorded points, and subsequently averaged over the duration of the pilot project. No localization errors were detected in the recorded trajectories. The geolocation analysis allowed the identification of the paths taken daily by the rider and the calculation of the average travel speed maintained throughout the entire experimental period (Figure 9a). A heatmap of the delivery routes was also generated using the vector data, in order to identify the most frequently traveled paths during the monitoring campaign. This visualization supports the analysis of route patterns and highlights areas of repeated use within the urban delivery network (Figure 9b). By cross-referencing this information with the locations of commercial activities in the urban center, a strong concentration of deliveries was observed along central shopping streets and at the logistics hub where goods are transferred.

By integrating the data collected by the sensor with the corresponding road segments, it was also possible to calculate the average speed during the experiment. An average speed of 10.5 km/h was recorded when excluding stops, and 7.7 km/h when including delivery times. In terms of travel distances, an average of 10.5 km per day was observed, corresponding to approximately 200 km over the entire study period. Secondly, once the calibration procedure was validated, it was applied to the data collected by the device on the routes in the city of Faenza during the pilot period. Using the GPS data collected by the device, the results of the bicycle routes could be mapped.

**Figure 10:**
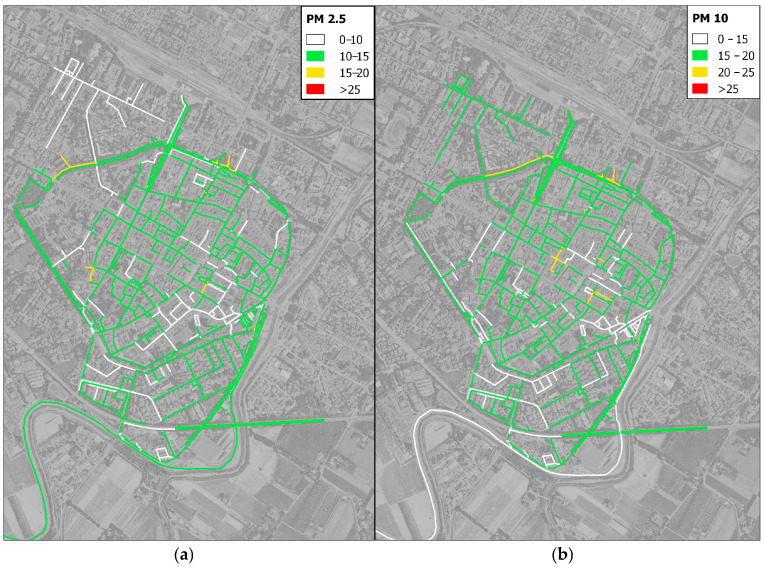
Environmental map in Faenza urban center: PM2.5 (**a**) and PM10 (**b**).

Below are the mappings of the primary pollutants collected by the environmental sensors during the survey (Figure 10). It is important to acknowledge that the results should be regarded as preliminary. This is due to the fact that they are based solely on the data collected over a period of 19 days, and it is possible that they are influenced by particular conditions associated with the limited duration of the monitoring period.

First and foremost, the integration of the environmental sensor on the cargo bike enabled good spatial coverage of the urban center. The average values collected over the monitoring period were assigned to each link within the city center network to assess the corresponding levels of environmental pollution. It was observed that, during most delivery routes, the air quality index ranged between level 1 (good) and level 2 (satisfactory), indicating a generally low presence of particulate matter in the historic center. Indeed, PM concentrations typically remained below 20 µg/m^3^, with the exception of more heavily trafficked urban roads. Values exceeding 25 µg/m^3^ were recorded only in the northern part of the city, where traffic density is significantly higher than in the central areas. A few potentially critical segments within the urban core were also identified; these may warrant further investigation—possibly through an expanded sample size—as they could be influenced by localized or temporary factors.

## 4. Discussion

The results, between the particulate matter values obtained from mobile sensors and fixed ones, demonstrate a strong linear correlation in terms of R^2^, for PM2.5 (R^2^ = 0.794) and PM10 (R^2^ = 0.616). However, as previous studies have shown [23,52], only high linear correlation is not sufficient to fully validate the proper functioning of the sensor. Therefore, in order to calibrate the mobile sensor, the data were evaluated using modelling techniques commonly found in the literature [25,26]. This analysis led us to test the different predictive models for correcting sensor measurements. For this reason, the observations underscore the validity of employing the MLR statistical analysis model. The MLRs’ outcomes show the good explanatory power of the models (R^2^ = 0.810 for PM2.5 and R^2^ = 0.645 for PM10) and exhibit no concerning autocorrelation issues. Compared to findings in the literature on this subject, the results obtained for the tested LCSs were similar [27,29]. Complementarily, while robust regression provides resilience against anomalous data points, it does not necessarily improve the overall predictive accuracy as measured by RMSE and MAE. The multiple linear model remains preferable when the goal is to minimize average prediction errors across the collected dataset. However, the data cover only two months for the calibration of the fixed station and the mobile sensors. Although this period did not allow for an optimal evaluation of the sensors to be conducted, it remains consistent with the timeframes adopted in similar studies [33,36,41]. Future works could increase the environmental sample to add diagnostic plots and residual analyses for elucidating model behavior under varying data conditions. In the second part of the research, the Cargo-Logistic Project of Faenza proved to be the perfect candidate for testing the sensors. This kind of project is well represented in the literature. However, it frequently exhibits an absence of detailed spatial mapping of urban pollution patterns. Consequently, the integration of GPS and environmental sensing technologies on the cargo bike enabled a spatial analysis of delivery activity and air quality in Faenza’s urban center. The GIS analysis of collected routes revealed consistent daily coverage, with a concentration of deliveries along central commercial streets and near the logistics hub. The ob6tained environmental data indicated a generally satisfactory air quality index, with low levels of particulate matter (mostly <20 µg/m^3^). Only a few critical streets with high levels of traffic exceeded 25 µg/m^3^. These hotspots may warrant further monitoring; however, overall, the results confirm a low potential for pollutant exposure for cargo bike deliveries within the urban area.

The findings of this study are consistent with those of previous research in the relevant literature [23,41] and they also demonstrate the feasibility of using mobile sensors for the purpose under consideration, with a high degree of approximation. Scientific progress in the development of such LCSs, which is becoming increasingly economical and compact, highlights the numerous prospects of integrating these instruments into a more complete monitoring network. Notwithstanding the favorable outcomes observed in this study, its findings are constrained by the inherent limitations of the data. It is evident that the data provided by the fixed reference station is subject to averaging over the course of a day, thereby precluding the possibility of conducting a detailed breakdown of the data. For instance, the pollutant collection with hourly resolution would have facilitated the establishment of a substantially more extensive database for comparison purposes. Moreover, it would enable a comprehensive assessment of the instruments’ sensitivity with respect to the daily fluctuations in pollutant levels, which is an issue in urban areas especially [52] due to anthropogenic activities. The possibility of having disaggregated data would support the use of predictive models, such as neural networks or decisional trees [53,54].

## 5. Conclusions

From a sustainability perspective, the employment of cargo bikes for last-mile deliveries is a valid alternative to conventional motorized vehicles in the logistics sector. However, while cargo cycling supports environmental and operational goals, it also raises concerns about the potential exposure of riders to elevated levels of air pollutants (especially fine particulate matter) during delivery operations in high-traffic zones. It is vital to consider the applicability of mobile sensors on bicycles for monitoring PM2.5 and PM10 levels. The final purpose of this research is to explore this possibility. A comparison was made of the data for these pollutants obtained through a mobile sensor and those from a fixed monitoring station belonging to the regional monitoring network. The consistency of the two measurements was validated by comparing a dataset comprising 49 days of observations, mainly through multiple linear regression (R^2^ = 0.810 for PM2.5 and R^2^ = 0.654 for PM10) and robust regression (R^2^ = 0.730 for PM2.5 and R^2^ = 0.630 for PM10). From the results issued, it was noted that the mobile stations seem to better assimilate the values of PM2.5 than the values of PM10. Moreover, as expected, humidity and temperature levels have an effect on the quality of the monitored data. To enhance this kind of analysis, for future studies, the authors suggest that data should be considered on an hourly basis (when applicable), as opposed to daily averages.

Following a thorough assessment of the data’s consistency, the sensors were employed for a period of 19 days in the municipality of Faenza, Italy, for last-mile deliveries by cargo bike. The obtained data were then utilized to create heat maps, which delineated the most frequented routes by the cargo bikers and the PM levels on the tracks. The results obtained from this study are presented with the aim of establishing groundwork for integrating commercial bicycle-mounted instruments that are capable of providing real-time feedback to riders regarding the environmental conditions they are in. This would enable cyclists to make informed route choices based not only on cost-efficiency but also on their level of exposure to air pollutants. Future research on the subject could explore route choice according to awareness of pollutant exposure; it could make use of simulation software or dedicated surveys. A large-scale integration of mobile sensors to the official monitoring network has the potential to provide a much more branched and widely covered return of pollutants, temperature, humidity, and noise levels. The integration of such data with official stations certainly has the potential to increase pollution mapping strategies at a local planning level. This information could then be shared with the municipal authorities to both inform citizens about the current environmental conditions and support the development of targeted strategies to address urban air quality issues.

## Figures and Tables

**Table 1:** Smart Citizen Kit sensor specifications.

Sensors	Measurement	Units	Sampling Frequencies
Sensirion SHT-31	Air temperature	°C	4 Hz
	Relative humidity	%RH	
Invensense ICS-434342	Noise level	dBA	50 Hz to 20 kHz
Rohm BH1721FVC	Ambient light	lx	50 Hz
NXP MPL3115A26	Barometric pressure	kPa	128 Hz
AMS CCS811	Equivalent carbon dioxide	ppm	60 samples/second
	Volatile organic compounds	ppb	
Plantower PMS 5003	Particulate matter (PM1, PM2.5, PM10)	µg/m^3^	1–2 Hz
NEO-M8U GPS	Position	Lat, Long	30 Hz

**Table 2:** Italian threshold for particle matter (**a**) and air quality index (**b**).

(**a**)		
**Pollutant**	**Time**	**Threshold**
PM10	Yearly	40 µg/m^3^
	Daily	50 µg/m^3^
PM2.5	Annual	25 µg/m^3^
(**b**)		
**PM2.5**	**PM10**	**Index**
0–10	0–15	Good
10–15	15–20	Satisfactory
15–20	20–25	Moderate
>25	>25	Poor

**Table 3:** Variables employed in the model.

Variable	Description	Unity of Measure
Fix_Sensor_PM25	Daily average particulate PM2.5 value gathered by the fixed sensor	µg/m^3^
Mobile_Sensor_PM25	Daily average particulate PM2.5 value gathered by a mobile sensor	µg/m^3^
Fix_Sensor_PM10	Daily average particulate PM10 value gathered by the fixed sensor	µg/m^3^
Mobile_Sensor_PM10	Daily average particulate PM10 value gathered by a mobile sensor	µg/m^3^
Humidity	Relative humidity gathered by a mobile sensor	%
Temperature	Temperature gathered by a mobile sensor	°C

**Table 4:** Pearson’s correlation matrix between environmental variables and PM concentrations (*n* = 49).

		Temperature	Humidity	Mobile_Sensor_PM25	Mobile_Sensor_PM10	Fix_Sensor_PM25	Fix_Sensor_PM10
**Temperature**	Pearson’s cor.	1.000	−0.794 **	−0.731 **	−0.734 **	−0.655 **	−0.505 **
**Humidity**	Pearson’s corr.	−0.794 **	1.000	0.593 **	0.591 **	0.454 **	0.308 *
**Mobile_Sensor_PM25**	Pearson’s cor.	−0.731 **	0.593 **	1.000	1.000 **	0.891 **	0.784 **
**Mobile_Sensor_PM10**	Pearson’s cor.	−0.734 **	0.591 **	1.000 **	1.000	0.893 **	0.785 **
**Fix_Sensor_PM25**	Pearson’s cor.	−0.655 **	0.454 **	0.891 **	0.893 **	1.000	0.938 **
**Fix_Sensor_PM10**	Pearson’s cor.	−0.505 **	0.308 *	0.784 **	0.785 **	0.938 **	1.000

**. The correlation is significant at the level of 0.01 (two-tailed). *. The correlation is significant at the level of 0.05 (two-tailed).

**Table 5:** MLR for PM25 model recap.

R	R-Squared	Adjusted R-Squared	Std. Error	DW
0.900 ^a^	0.810	0.797	5.552	1.989

^a^ Predictors: humidity, temperature, Mobile_Sensor_PM25.

**Table 6:** ANOVA for PM2.5.

Model	Sum of Squares	Mean Square	F	Sign.
Regression	5904.492	1968.164	63.853	<0.001 ^b^
Residual	1387.059	30.824		
Total	7291.551			

^b^ Dependent variable: Fix_Sensor_PM25.

**Table 7:** MLR for PM10 model recap.

R	R-Squared	Adjusted R-Squared	Std. Error	Durbin–Watson
0.809 ^a^	0.645	0.631	7.767	1.519

^a^ Predictors: humidity, temperature, and Mobile_Sensor_PM10.

**Table 8:** ANOVA for PM10.

Model	Sum of Squares	Mean Square	F	Sign.
Regression	5134.689	1711.563	28.372	<0.001 ^b^
Residual	2714.568	60.326		
Total	7849.347			

^b^ Dependent variable: Fix_Sensor_PM10.

**Table 9:** Performance statistical indices for the calibration.

	MLR		RRC	
	PM2.5	PM10	PM2.5	PM10
**R^2^**	0.810	0.645	0.730	0.630
**RMSE**	6.261	7.443	6.261	7.769
**MAE**	3.382	5.393	3.810	5.346

## Data Availability

The dataset presented in this study is partially available on request.

## Abbreviations

The following abbreviations are used in this manuscript:

MLR	multiple linear regression
PM	particulate matter
PM2.5	particulate matter 2.5
PM10	particulate matter 10
LCSs	low-cost sensors
RR	robust regression
RANSAC	Random Sample Consensus
